# Determination of Incipient Tuberculosis with the Aid of Roentgen Rays

**Published:** 1898-07

**Authors:** J. Preston Hoskins

**Affiliations:** Princeton, N. J.


### ABSTRACTS FROM FOREIGN JOURNALS.

### GERMAN.

Under the Direction of J. Preston Hoskins,
PRINCETON, N. J.

Determination of Incipient Tuberculosis with the aid of Roentgen Rays. Among the many attempts which have been made to utilize the Roentgen rays in medicine, probably those are the most important which Kelsch and Boinon, of Lyons, have lately described before the Paris Academy of Medicine. It is an attempt to determine the presence of tubercles in the early stage of the disease. These physicians have for months been investigating the chests of young people with the radioscope. In their investigations the patient is always examined from the back side of the trunk, because a clearer view can be obtained here than from the front.

The physicians describe the view as very striking which the thoracic cavity makes upon the cyanide of potassium screen; everything lives and moves upon the same. In a healthy man the lungs are transparent from top to bottom; expiration is visible in the rising and sinking of the ribs, the beating of the heart is easily recognized, as well as the bending of the aorta and the movement of the diaphragm, which on expiration rises to the sixth rib, and on inspiration sinks to the eighth or ninth—*i. e.*, at every breath it moves eight to ten centimetres, and resembles a mighty pump in its action.

After the physicians, by continued observation, had familiarized themselves with this image of the thoracic cavity, they endeavored to discover in it the signs of tuberculous disease. The investigation covered 124 persons who, for various reasons, had been admitted to the hospital, but in whom no signs of tuberculous disease of the lungs could be discovered by the ordinary means. In fifty-one cases various deviations from the normal condition of the lungs were observable; a decline in the transparency of one or both apices of the lungs, a one-sided decrease in the movement of the diaphragm, abnormal condition of the air-bubbles on one or on both sides. Since the apices of the lungs, the ends of the windpipe, and the pleura form especially the centre of tuberculosis, the investigators concluded that the observations in regard to the changes in these organs were to be regarded as signs of an early stage of tuberculosis which heretofore could not be detected. In five cases which were thus examined the presence of tuberculosis was confirmed by a postmortem examination.

With confidence in the reliability of such observations, the members of the academy present agreed that the early diagnosis of tuberculous diseases of the lungs was the most valuable service which the Roentgen rays had up till now performed for medicine. Reference was immediately made to the great importance which this method of investigation would have in the examination of recruits, since the germs of consumption often develop in military service under the continued hardships. Since tuberculous persons often have a very healthy appearance, up till now there has been no means of rejecting persons with lung-diseases before mustering them in. The editor would here rather leave the last question open in regard to the possibility of determining tuberculous conditions in persons who arouse no suspicion of the disease on account of their healthy appearance.—*Thierärztliches Centralblatt*, March 1, 1898.

